# Small Spatial Scale Drivers of Secondary Metabolite Biosynthetic Diversity in Environmental Microbiomes

**DOI:** 10.1128/msystems.00724-22

**Published:** 2023-02-15

**Authors:** Aileen Ute Geers, Mikael Lenz Strube, Mikkel Bentzon-Tilia

**Affiliations:** a Department of Biotechnology and Biomedicine, Technical University of Denmark, Lyngby, Denmark; University of Sao Paulo

**Keywords:** antibiotics, microbiomes, nonribosomal peptides, polyketides, secondary metabolites

## Abstract

In the search for novel drug candidates, diverse environmental microbiomes have been surveyed for their secondary metabolite biosynthesis potential, yet little is known about the biosynthetic diversity encoded by divergent microbiomes from different ecosystems, and the environmental parameters driving this diversity. Here, we used targeted amplicon sequencing of adenylation (AD) and ketosynthase (KS) domains along with 16S sequencing to delineate the unique biosynthetic potential of microbiomes from three separate habitats (soil, water, and sediments) exhibiting unique small spatial scale physicochemical gradients. The estimated richness of AD domains was highest in marine sediments with 656 ± 58 operational biosynthetic units (OBUs), while the KS domain richness was highest in soil microbiomes with 388 ± 67 OBUs. Microbiomes with rich and diverse bacterial communities displayed the highest PK potential across all ecosystems, and on a small spatial scale, pH and salinity were significantly, positively correlated to KS domain richness in soil and aquatic systems, respectively. Integrating our findings, we were able to predict the KS domain richness with a RMSE of 31 OBUs and a *R*^2^ of 0.91, and by the use of publicly available information on bacterial richness and diversity, we identified grassland biomes as being particularly promising sites for the discovery of novel polyketides. Furthermore, a focus on acidobacterial taxa is likely to be fruitful, as these were responsible for most of the variation in biosynthetic diversity. Overall, our results highlight the importance of sampling diverse environments with high taxonomic diversity in the pursuit for novel secondary metabolites.

**IMPORTANCE** To counteract the antibiotic resistance crisis, novel anti-infective agents need to be discovered and brought to market. Microbial secondary metabolites have been important sources of inspiration for small-molecule therapeutics. However, the isolation of novel antibiotics is difficult, and the risk of rediscovery is high. With the overarching purpose of identifying promising microbiomes for discovery of novel bioactivity, we mapped out the most significant drivers of biosynthetic diversity across divergent microbiomes. We found the biosynthetic potential to be unique to individual ecosystems, and to depend on bacterial taxonomic diversity. Within systems, and on small spatial scales, pH and salinity correlated positively to the biosynthetic richness of the microbiomes, Acidobacteria representing the taxa most highly associated with biosynthetic diversity. Ultimately, understanding the key drivers of the biosynthesis potential of environmental microbiomes will allow us to focus bioprospecting efforts and facilitate the discovery of novel therapeutics.

## INTRODUCTION

Microbial secondary metabolites are likely the most important source of inspiration for the therapeutic small molecules developed over the past 4 decades (1). Specifically, the discovery of microbial secondary metabolites with antibacterial activity has, since their discovery in the 1920s, advanced medicine and human health tremendously, saving countless lives over the last century. However, these advances are threatened by the increasing occurrence of antimicrobial resistance (AMR) worldwide. In 2019, around half of the urinary tract infections caused by Escherichia coli or Klebsiella pneumonia were reported to be resistant to the first-line antibiotic co-trimoxazole (2), and collectively bacterial AMR was associated with close to five million deaths in 2019 (3). To overcome this crisis, action has to be taken including limiting the administration of antibiotics and securing a continuous supply of novel anti-infective agents (4).

Historically, novel antibiotics were discovered by screening the activity of isolated environmental strains against pathogens using agar overlay inhibition assays (5). Over the decades, increasing re-discovery rates turned this approach inefficient, and the potential of cultured microbes was deemed exhausted (6). However, recent advances in high-throughput sequencing have revealed an astounding biosynthetic capacity of natural microbiomes to produce chemical diversity (7–9), paving the way for a deeper understanding of the distribution and diversity of the biosynthesis potential of natural microbiomes. Two major classes of secondary metabolites with a range of clinically relevant bioactivities are the nonribosomal peptides (NRPs) and the polyketides (PKs), and multiple compounds of these classes are currently in use as antibiotics, e.g., the NRP vancomycin and the PK erythromycin. Both NRPs and PKs are produced by large multimodular synth(et)ases, where each module adds one building block to the growing product chain, usually acetyl-coenzyme A or its analogs in the case of PKs, or amino acids in the case of NRPs. These mega enzymes consist of several conserved domains, e.g., adenylation domains (AD) of nonribosomal peptide synthetases (NRPSs) and ketosynthase (KS) domains of polyketide synthases (PKSs), which have been most widely targeted for amplicon sequencing to approximate the biosynthetic potential, and its diversity in environmental microbiomes (8). Through AD and KS amplicon sequencing, thousands of unique operational biosynthetic units (OBUs) have been detected in soil, underlining the large unexplored potential of the immensely diverse and predominantly uncultured microbial communities residing in this niche (10–13). Yet, in order to focus bioprospecting efforts on the most promising microbiomes, a more comprehensive understanding of the drivers of this natural product diversity is needed (8). Dispersal limitation, or geographic distance, soil depth, and ‘biome’ type have previously been reported as the strongest drivers of biosynthetic diversity in soil on a continent-wide scale (9, 12, 13), while small spatial scale drivers have not yet been identified. Similarly, marine surface water and marine sediments have been reported to be rich reservoirs of PK and NRP biosynthetic genes, displaying significant differences in composition and degree of novelty compared to soil (7, 14). However, environmental drivers of this potential have not been investigated, and general patterns across diverse systems have hence not yet been identified.

We speculated that the biosynthesis potential, i.e., biosynthetic domain richness, of environmental microbiomes is dependent on the bacterial and fungal community composition, which, in turn, may be determined by specific environmental variables. We therefore set out to investigate the natural product biosynthesis potential of proximate microbiomes from three separate systems, each of which exhibited physicochemical gradients likely to affect the microbial community composition, i.e., pH (soil system), salinity (aquatic system), and redox potential (marine sediment system) (15–18). Additionally, we measured a range of auxiliary variables, and investigated bacterial and fungal taxonomic diversity, with the ultimate goal of predicting the biosynthesis potential in microbiomes using readily quantifiable parameters.

## RESULTS

**FIG 1:**
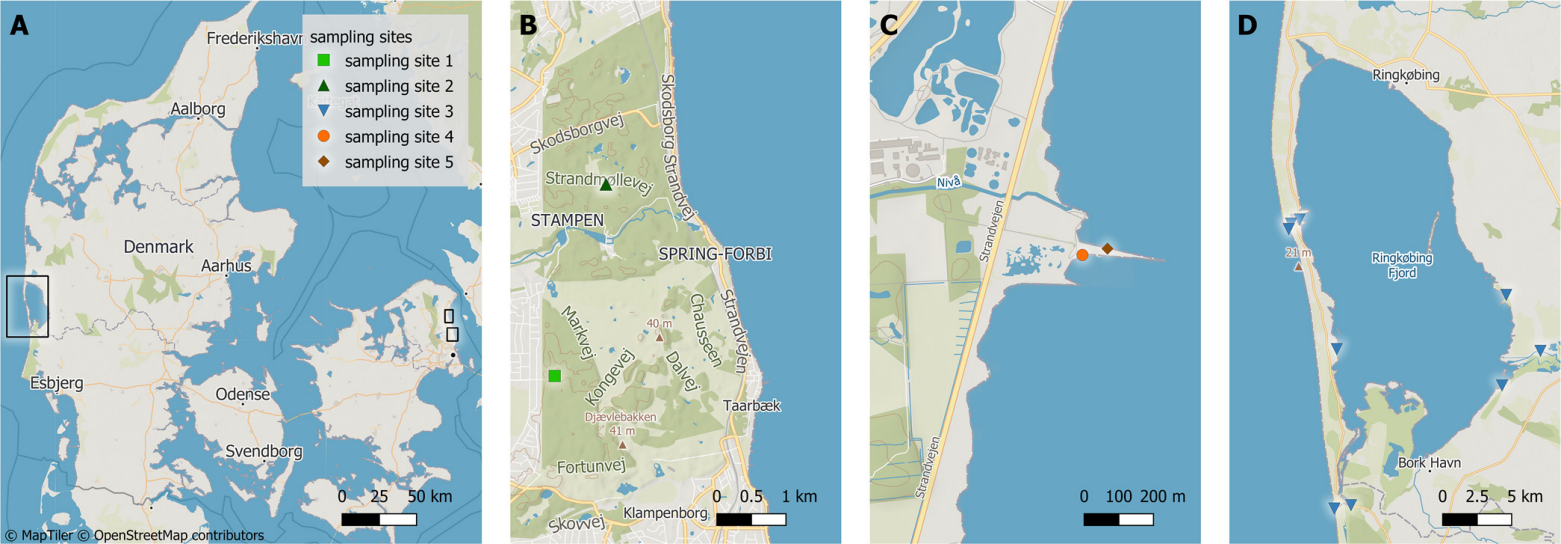
Geographic location of sampling sites marked with black squares (A). Precise locations of soil sampling site 1 and 2 in Dyrehaven (B, green square and triangle), of sediment sampling sites 4 and 5 in Nivå bay (C, orange circle and brown diamond), and of aquatic sampling site 3 in Ringkøbing Fjord (D, blue triangles).

**FIG 2:**
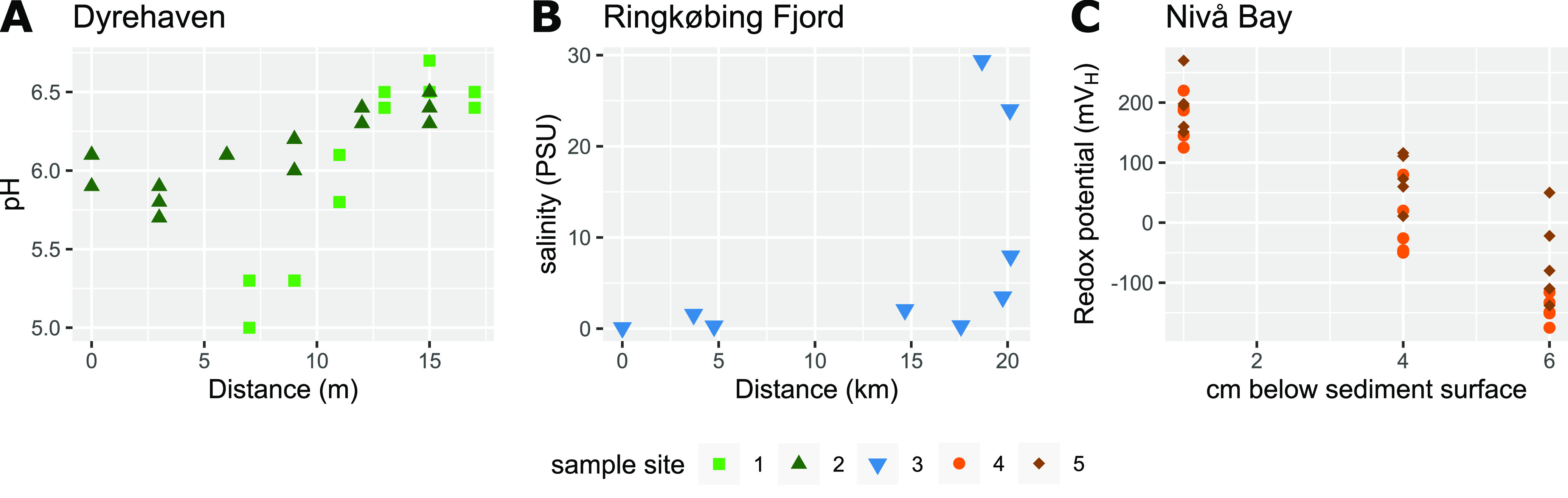
Profile of predominant physicochemical gradients in each ecosystem. (A) The pH of the topsoil at sampling site 1 (green square) and 2 (green triangle) in Dyrehaven along the sampled transects, with distance given in relation to the center of the closest pine grove. (B) Measured salinity in and around the Ringkøbing Fjord (sample site 3, blue triangles) versus distance in km from the Skjern Å river outlet. (C) The redox potential of the sediment at sampling site 4 (orange circle) and site 5 (brown diamond) at Nivå bay at three different depths below the sediment surface.

To expose environmental parameters driving the biosynthesis potential of natural microbiomes, we analyzed microbial community DNA from sampling sites dispersed across small spatial scales in three contrasting systems exhibiting different physicochemical gradients, i.e., a forest top soil system, a marine coastal sediment system, and an aquatic surface system (Fig. 1). In forest soil, we observed pH gradients from 5 to 6.7 across transects (Fig. 2A). pH was highly correlated to carbon content and, to a lesser degree, to other parameters, including sodium, sulfate, and phosphate (*R* = 0.8, −0.68, 0.77, 0.75) (Table S1). As expected (19), vertical sediment cores collected at sites S4 and S5 in Nivå Bay exhibited steep redox gradients within the top 7 cm, ranging from 270 mV_H_ in the oxygenated surface sediments to -175 mV_H_ in the lower layers (Fig. 2C). The redox potential correlated significantly with the moisture content of the sediment (*R* = 0.61). In the aquatic system, a salinity gradient was observed across the sampling stations ranging from 0 PSU in the Skjern Å river outlet up to almost full salinity (30 PSU) in the North Sea outside the Ringkøbing Fjord estuary (Fig. 2B). Naturally, specific environmental parameters, such as the concentrations of sodium and other ions, were positively correlated with salinity (*R* = 0.995) (Table S1). Bacterial abundances ranged from 2.6 × 10^6^ ± 1.2 × 10^6^ cells mL^−1^ in surface water, over 7.5 × 10^6^ ± 3.8 × 10^6^ cells g^−1^ in sediments, to 3.4 × 10^8^ ± 2.5 × 10^8^ cells g^−1^ in soil. The abundances observed in soil were significantly correlated with aluminum concentrations (*R* = 0.59) (Table S1).

10.1128/msystems.00724-22.1 TABLE S1 Measured physicochemical metadata across all systems, and additionally estimated bacterial and fungal diversity measures. Download Table S1, XLSX file, 0.03 MB. Copyright © 2023 Geers et al. 2023 Geers et al. https://creativecommons.org/licenses/by/4.0/ This content is distributed under the terms of the Creative Commons Attribution 4.0 International license.

### Taxonomic and secondary metabolite biosynthetic diversity across systems.

**FIG 3:**
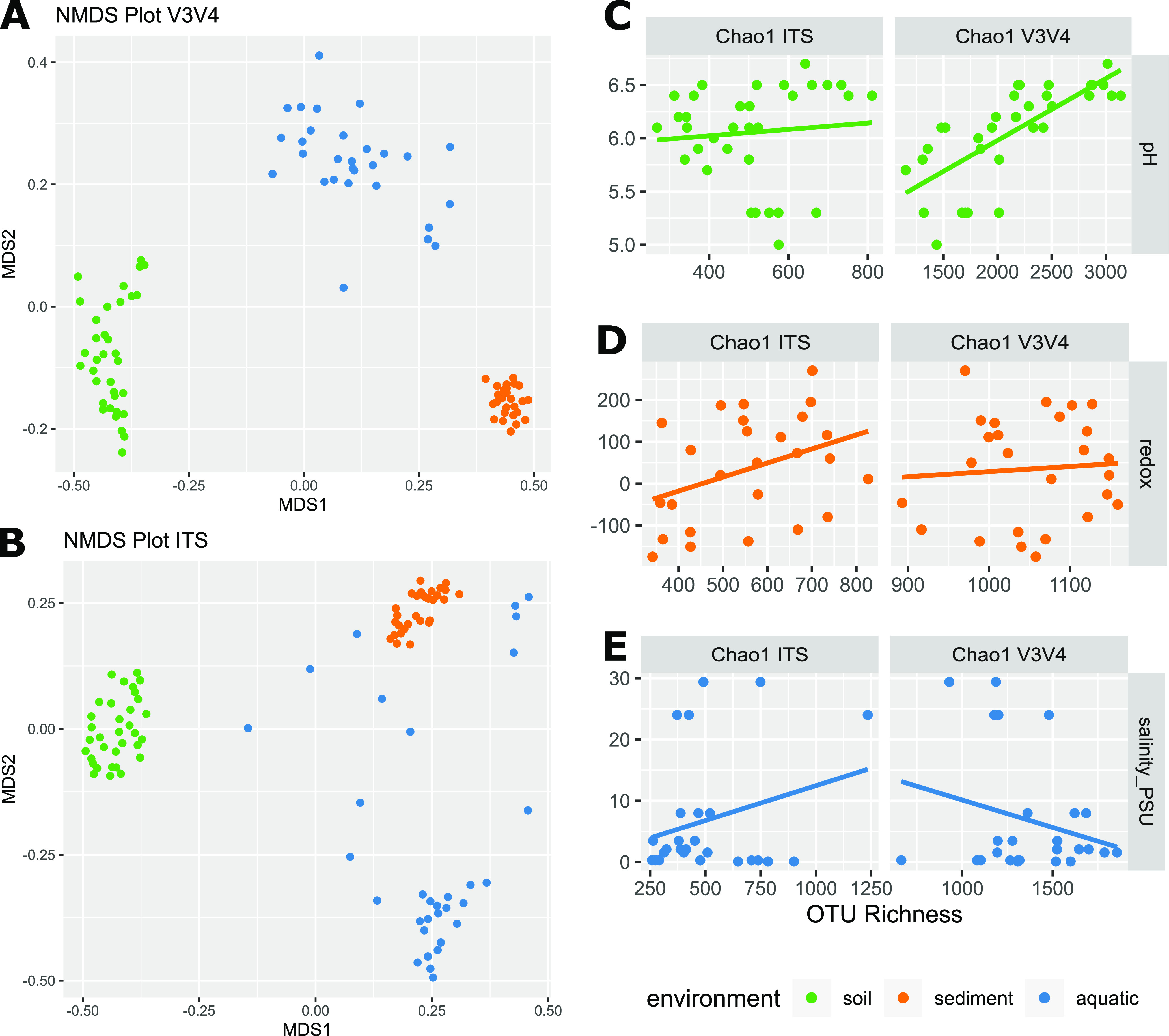
Bacterial and fungal community diversity, and their relation to predominant physicochemical gradients. (A and B) Beta-diversity of the bacterial and fungal taxonomic community composition depicted by non-metric multi-dimensional scaling plots. Soil samples in green, sediment in orange, and aquatic in blue. (C) Estimated fungal and bacterial Chao1 richness in the soil microbiome as a function of pH. (D) Estimated fungal and bacterial Chao1 richness in the sediment microbiome versus the redox potential mV_H._ (E) Estimated fungal and bacterial Chao1 richness in the aquatic microbiomes versus the salinity PSU. Linear Pearson correlations are indicated with a solid line.

Bacterial and fungal communities clearly differed across systems (PERMANOVA, *P* = 0.001; Fig. 3A and B). The proteobacterial phylum was highly represented (24.7 to 48.3%) across all three systems and dominated in the two aquatic environments, while the dominating phyla in the soil system were Acidobacteria (24.7%) and Actinobacteria (16.2%) (Fig. S1). In addition to Proteobacteria, the Bacteriodetes (11.9% and 20.8%) and Cyanobacteria (13.2% and 2.8%) were abundant in sediments and surface waters, respectively. Fungal soil communities were dominated by the Ascomycota (42%) and Basidiomycota (42.2%), followed by Zygomycota (8.69%), while the majority of fungal zOTUs in the aquatic systems could not be assigned to a known phylum (Fig. S2). The soil had the richest and most diverse bacterial community with an estimated number (Chao1) of 2130 ± 557 zOTUs per sample, followed by the aquatic system and the marine sediment with 1354 ± 279 zOTUs and 1054 ± 73 zOTUs, respectively. Fungal communities exhibited similar patterns of alpha diversity across all sampled environments with Chao1 richness estimates of 520 ± 170 zOTUs per sample (Fig. S3).

10.1128/msystems.00724-22.5 FIG S1 Relative abundance of bacterial phyla based on the V3V4 zOTUs in each sample. The 11 most abundant phyla are colored in, the remaining are grouped together into ‘other’, unclassified are denoted as ‘NA’. Download FIG S1, EPS file, 0.2 MB. Copyright © 2023 Geers et al. 2023 Geers et al. https://creativecommons.org/licenses/by/4.0/ This content is distributed under the terms of the Creative Commons Attribution 4.0 International license.

10.1128/msystems.00724-22.6 FIG S2 Relative abundance of fungal phyla based on the ITS zOTUs in each sample. The 3 most abundant phyla are colored in, the remaining are grouped together into ‘other’, unclassified are denoted as ‘NA’. Download FIG S2, EPS file, 0.1 MB. Copyright © 2023 Geers et al. 2023 Geers et al. https://creativecommons.org/licenses/by/4.0/ This content is distributed under the terms of the Creative Commons Attribution 4.0 International license.

10.1128/msystems.00724-22.7 FIG S3 Box-and-whisker plots of estimated fungal (ITS) and bacterial (V3V4) Chao1 richness for each ecosystem. Download FIG S3, EPS file, 0.1 MB. Copyright © 2023 Geers et al. 2023 Geers et al. https://creativecommons.org/licenses/by/4.0/ This content is distributed under the terms of the Creative Commons Attribution 4.0 International license.

For the bacterial fraction of the soil microbiomes, the main drivers of community structure were sampling site and pH, collectively explaining 39% of the observed variation (PERMANOVA, *P* = 0.001), and simultaneously a strong and significant positive correlation between bacterial richness (Chao1) and pH was observed (*R* = 0.73, *P*_adj_ = 6.99 × 10^−5^) (Fig. 3C). Other covariates describing bacterial taxonomic richness where distance across the transect and concentrations of iron, magnesium, phosphate, and sulfate (*R* = 0.78, −0.54, 0.67, −0.72, 0.56). The fungal fraction of the soil microbiomes did not exhibit similar pH-dependent patterns (Fig. 3C), though fungal richness was significantly correlated with iron content (*R* = −0.63, *P*_adj_ = 0.013).

Sediment microbiomes were most strongly affected by distance as the grouping based on sampling sites explained 31% of the observed bacterial variation (*P* = 0.001), while the redox potential could explain an additional 8% (*P* = 0.005). Fungal communities exhibited similar patterns with 42% of the variation being explained by sampling site. Species richness was not correlated with any of the measured variables for bacteria or fungi (Fig. 3D).

In the aquatic environment, salinity was the main driver of community structure, explaining 39% and 23% of the variation in the bacterial and fungal communities, respectively. However, salinity did not affect the overall richness of the bacterial or fungal microbiomes (Fig. 3E).

**FIG 4:**
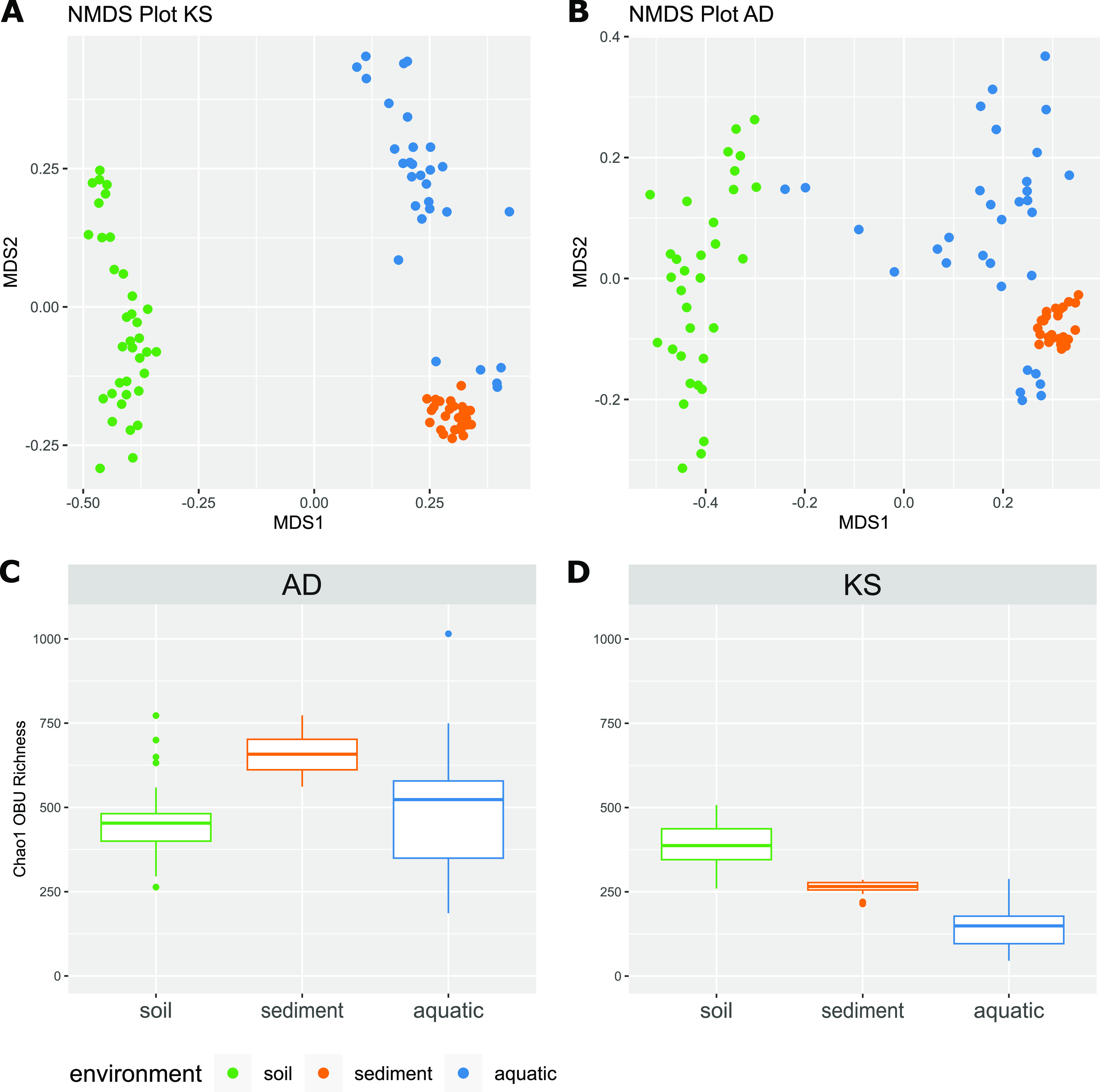
Diversity of AD and KS domains in different habitats. (A and B) Non-metric multi-dimensional scaling plots of the soil (green), sediment (orange), and aquatic (blue) samples, based on the Bray-Curtis dissimilarity matrix. (C and D) Box-and-whisker plots of estimated AD and KS domain Chao1 richness for each ecosystem. Box edges are the first and third quartiles, and whiskers are a maximum 1.5 times the interquartile range from the box edge.

As for the taxonomic diversity, the composition of functional genes involved in non-ribosomal peptide and polyketide biosynthesis, i.e., AD and KS domains, significantly differed between all 3 environments (PERMANOVA, AD *R*^2^ = 0.32 *P* = 0.001, KS *R*^2^ = 0.38 *P* = 0.001) (Fig. 4A and B), with sediment microbiomes exhibiting limited variation between samples compared to the other systems. Overall, we observed a considerable richness of AD (531 AD OBUs ± 158) and KS (281 KS OBUs ± 116) domains, with soil microbiomes being the richest in different KS domains (388 ± 67), and marine sediments exhibiting higher richness in AD domains (656 ± 58) (Fig. 4C and D).

Comparing the OBUs from each of the 3 systems with the curated MiBIG database of BGCs encoding characterized NPs showed that the vast majority of OBUs did not represent BGCs for which a known compound has been described in all three systems (Fig. S4). The highest relative abundance of matching AD OBUs was found in seawater with 0.8% ± 1%, whereas the highest relative abundance of matching KS OBUs was observed in soil (17% ± 15%). For sediments, the relative abundance of matching OBUs were 0.03 ± 0.04% and 0.03 ± 0.004% for AD and KS OBUs, respectively.

10.1128/msystems.00724-22.8 FIG S4 Relative abundance of AD OBUs (A) and KS OBUs (B), which matches BGCs with characterized compounds in MiBIG v3.1 in the 3 different systems. Download FIG S4, EPS file, 1.8 MB. Copyright © 2023 Geers et al. 2023 Geers et al. https://creativecommons.org/licenses/by/4.0/ This content is distributed under the terms of the Creative Commons Attribution 4.0 International license.

In order to investigate which bacterial strains were associated with changes in biosynthetic richness, we fitted a generalized linear model with lasso regularization using the centered log-transformed 16S zOTU table to either KS or AD domain richness (20). In the model, 15 bacterial zOTUs were enough to explain 90% of the variation in KS richness. The identified zOTUs were a good representation of the overall microbial community with a majority of Proteobacteria and Acidobacteria, followed by zOTUs from the phylum Verrucomicrobia, Cyanobacteria, Bacteroidetes, and Firmicutes (Table S2). By contrast, to explain 90% of the variation in AD richness, 47 bacterial zOTUs were needed, the majority of which were Proteobacteria, followed by Acidobacteria, Bacteroidetes, and Actinobacteria (Table S2).

10.1128/msystems.00724-22.2 TABLE S2 List of bacterial or fungal zOTUs that can explain 90% variation of KS or AD domain richness. Download Table S2, XLSX file, 0.01 MB. Copyright © 2023 Geers et al. 2023 Geers et al. https://creativecommons.org/licenses/by/4.0/ This content is distributed under the terms of the Creative Commons Attribution 4.0 International license.

### Cross-system correlations between metadata variables and biosynthesis potential.

**FIG 5:**
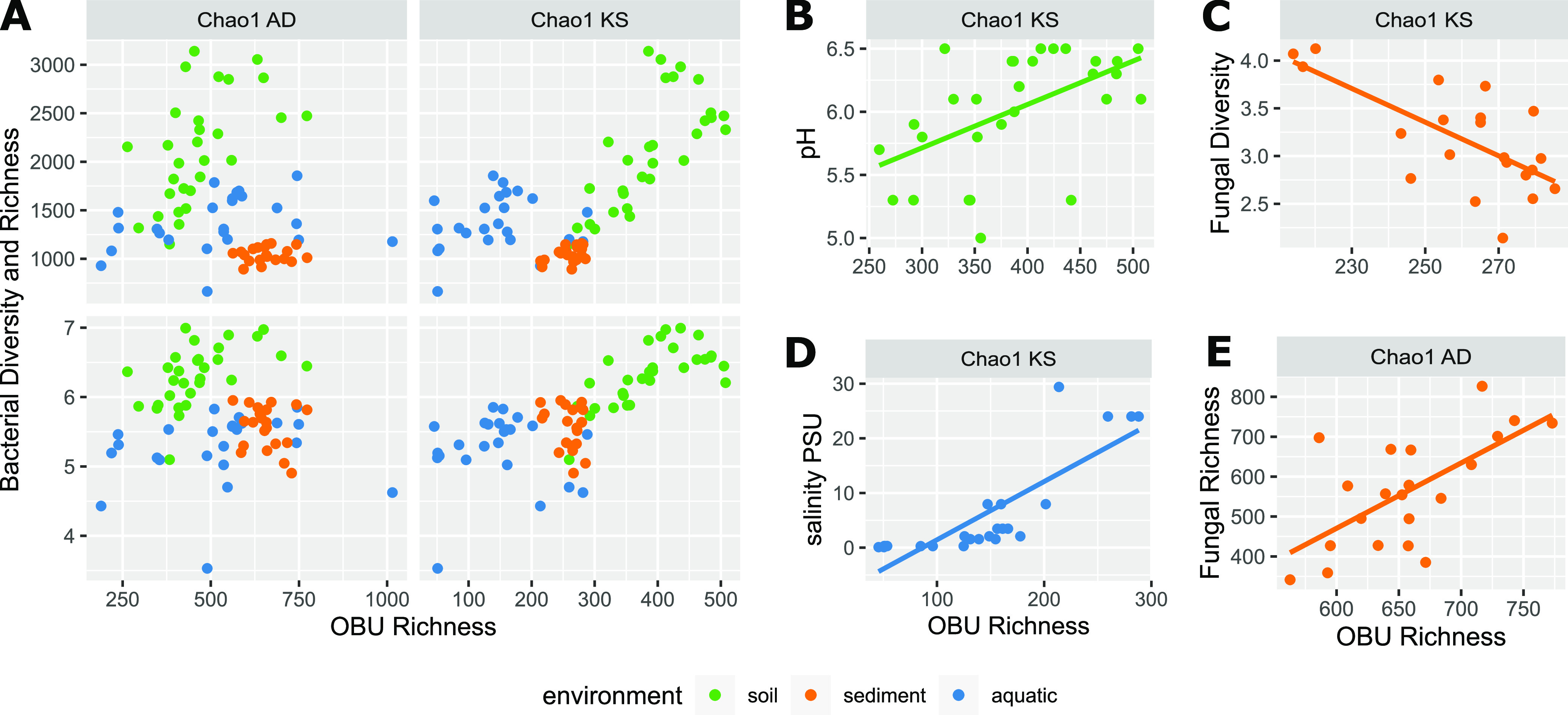
Linear correlations of AD and KS domain richness to taxonomic diversity and environmental variables. (A) Bacterial Chao1 richness and Shannon diversity in relation to biosynthetic Chao1 richness, colored according to the ecosystem: soil (green), sediment (orange), aquatic (blue). (B to E) KS domain or AD domain richness in relation to pH, salinity, fungal Shannon diversity, or fungal Chao1 richness in specific systems. Bold lines indicate the Pearson linear correlations.

To determine global drivers of the biosynthesis potential of microbiomes across systems, we looked for simple linear correlations between OBU richness and all measured metadata variables, including taxonomic diversity measures, and excluding parameters only determined for certain systems. Of 40 correlations, we focused on those with a significance of an adjusted *P* < 0.05 and R > |0.5|. For both the NRP and PK biosynthesis potential, we found a significant correlation to moisture content, though positive for NRPs and negative for PKs (Table 1). The NRP biosynthesis potential was negatively correlated with organic carbon content (LOI; *R* = −0.63, *P*_adj_ = 3.57 × 10^−6^), while the potential PK biosynthesis was negatively correlated with pH across systems (*R* = −0.69, *P*_adj_ = 3.99 × 10^−10^). Lastly, we found strong correlations between bacterial taxonomic diversity (Chao1 and Shannon) and KS richness (*R* = 0.68 *P*_adj_ = 3.83 × 10^−11^, *R* = 0.74 *P*_adj_ = 8.66 × 10^−14^), suggesting that a more diverse bacterial community results in a higher PK biosynthesis potential (Table 1 and Fig. 5A), while fungal diversity does not seem to influence the biosynthesis potential of neither NRPs nor PKs across systems.

**TABLE 1:** Significant linear correlations of KS or AD OBU Chao1 Richness to taxonomic and environmental variables across systems^*a*^

	Pearson correlations			
Variable	OBU Chao1	R	*P* value	*P*_adj_ value
Fe	KS	0.58	3.8 × 10^−9^	4.58 × 10^−8^
LOI	AD	−0.63	9.91 × 10^−8^	3.57 × 10^−6^
Moisture	AD	0.70	8.51 × 10^−10^	6.13 × 10^−8^
Moisture	KS	−0.76	7.75 × 10^−13^	1.86 × 10^−11^
NH3	KS	0.59	3.56 × 10^−5^	3.2 × 10^−4^
pH	KS	−0.69	2.77 × 10^−11^	3.99 × 10^−10^
S	KS	0.84	2.33 × 10^−14^	8.37 × 10^−13^
Shannon V3V4	KS	0.74	1.2 × 10^−15^	8.66 × 10^−14^
Chao1 V3V4	KS	0.68	2.13 × 10^−12^	3.83 × 10^−11^

a R and *P*-values are determined by Pearson correlations.

### System-specific correlations between metadata variables and biosynthesis potential.

We further investigated system-specific correlations between metadata variables and OBU richness, applying the same R and p cutoff values as before. In contrast to the cross-system observations, we observed a significant positive correlation between KS domain richness and soil pH (KS *R* = 0.51 *P*_adj_ = 0.030) (Fig. 5B). Additionally, the phosphate content and the magnesium concentration in soil were negatively correlated with KS richness (*R* = −0.62 *P*_adj_ = 0.0013, *R* = −0.57 *P*_adj_ = 0.0065) (Table S4). As observed across systems, the PK biosynthesis potential of soil microbiomes was positively correlated with bacterial richness and diversity (ChaoV3V4 *P*_adj_ = 9.1 × 10^−5^, ShannonV3V4 *P*_adj_ = 5.4 × 10^−4^) (Fig. 5A and Table S4). While the biosynthesis potential of the sediment microbiomes were, in general, very similar in their AD and KS composition and richness, these were affected by fungal diversity, whereas the PK biosynthesis potential in the aquatic system was significantly influenced by salinity and co-varying factors. (Fig. 5C to E, and Table S4).

10.1128/msystems.00724-22.4 TABLE S4 Significant linear correlations of (i) OBU richness to environmental variables and species diversity cross systems, (ii) OBU richness to environmental variables and species diversity system-specific, (iii) pH, redox potential, salinity, and cell abundances to other environmental variables system-specific, and (iv) bacterial and fungal species richness to environmental variables. Significance is determined by: adjusted *P* < 0.05 & R > |0.5|for all correlations. R and *P*-values are determined by Pearson correlations. Download Table S4, XLSX file, 0.01 MB. Copyright © 2023 Geers et al. 2023 Geers et al. https://creativecommons.org/licenses/by/4.0/ This content is distributed under the terms of the Creative Commons Attribution 4.0 International license.

### Predicting the biosynthesis potential of environmental microbiomes.

With the ultimate goal of predicting the capacity of a microbiome using readily available, or easily quantifiable variables, we built a generalized linear model and, after stepwise variable selection, we arrived at a specific model for KS richness estimation drawing upon system type and alpha diversity measures: log(KS) ~ system + log(Chao1_V3V4) + log(Shannon_V3V4). Testing the model on a subset of data, we reached a RMSE of 48.1 and a *R*^2^ of 0.72. With 280 ± 117 OBUs per sample, the error of the model is smaller than the standard deviation.

**FIG 6:**
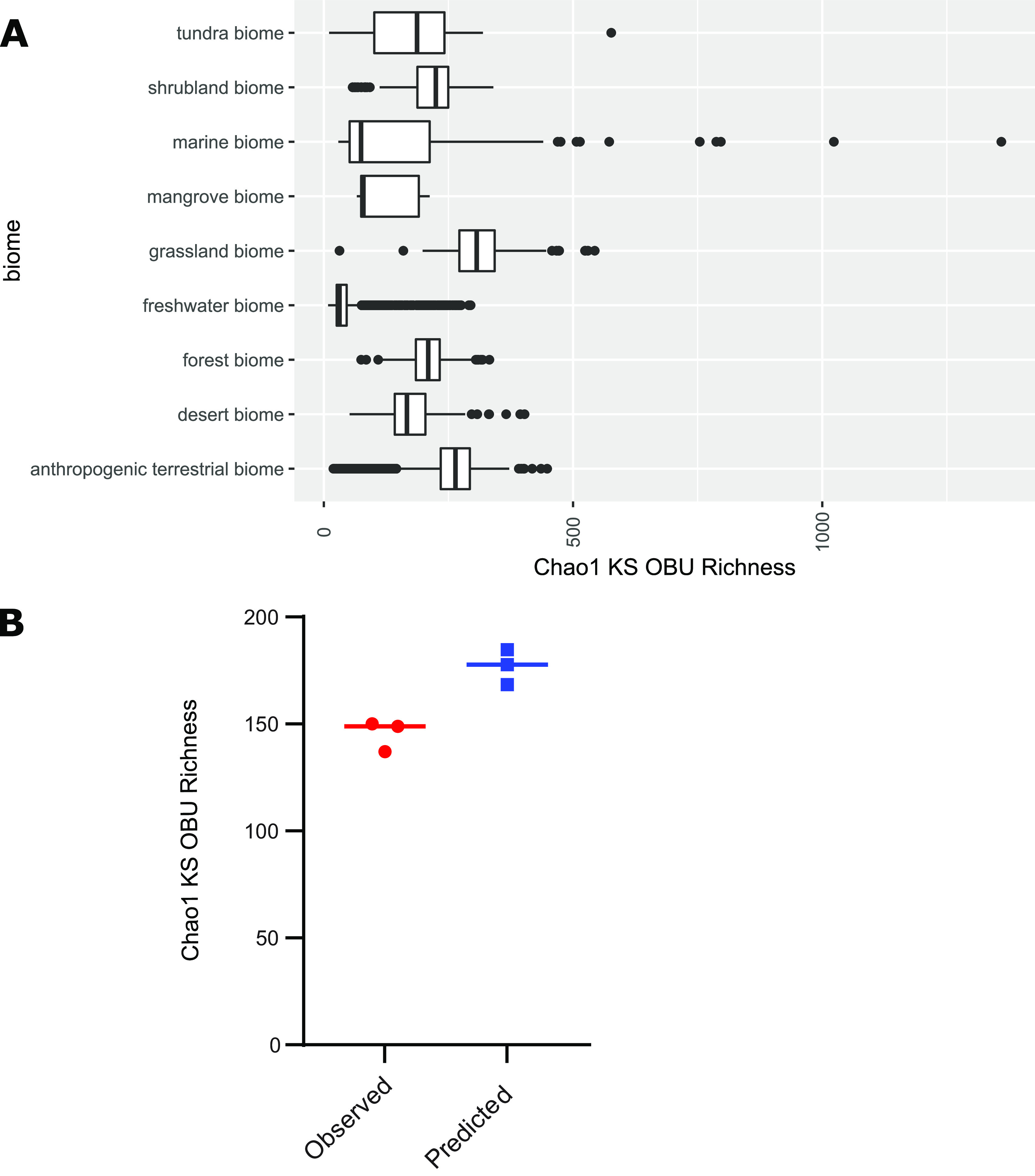
(A) Predicted KS domain Chao1 richness in different biomes from the Earth Microbiome data set. A generalized linear model was fit on measured bacterial richness, bacterial diversity, and ecosystem type (soil, sediment, saltwater, or freshwater) for predicting KS domain richness. (B) Predicted KS domain Chao1 richness using the model compared to actual KS domain Chao1 richness estimates generated from KS sequencing reads from the same triplicate seawater samples from (7).

Further accuracy of the model could be achieved by separating the water samples into freshwater and saltwater using a threshold of 1 PSU salinity. The improved model has a RMSE of 30.9 and a *R*^2^ of 0.91. Additionally, we tested if the model could be further generalized by excluding the system as a variable, and instead relying only on bacterial diversity and cell abundance as predictors for PK biosynthetic richness. However, this reduced the accuracy of the model resulting in a RMSE on the test data of 69.1, and a *R*^2^ of 0.561. It was not possible to build a model with an RMSE lower than the standard deviation for AD domains. We further applied our improved PK model on the Earth Microbiome data set (16), using the available bacterial richness, bacterial diversity, and the empo_3 classifications to predict the KS richness on a global scale. In contrast to our sediment samples from Nivå bay, sediment samples on a global scale (238 ± 102) seemed to be almost equally as rich in KS domains as soil samples (247 ± 62). Furthermore, the grassland biome stood out with a high mean PK biosynthetic richness of 312 ± 63 KS OBUs, while the highest individual predictions were observed in marine biomes (Fig. 6A). To further test the accuracy of our model, we predicted KS OBU richness based on bacterial diversity estimates from seawater (7), and compared the richness to the measured Chao1 KS OBU (Fig. 6B).

## DISCUSSION

An increasing number of studies using targeted sequencing of biosynthetic domains have revealed the vast, underexplored potential of the uncultured majority of microorganisms in the environment (7, 11–14, 21–23). Yet, differences in study design and the experimental approaches applied make it difficult to extrapolate findings across systems (8, 11), thus hampering efforts to identify global drivers of secondary metabolome diversity. Moreover, the environmental drivers of the biosynthetic diversity on a small spatial scale have been largely overlooked. Collectively, this has limited our ability to predict the biosynthetic diversity of environmental microbiomes. We found the microbial community composition and the biosynthetic potential to be highly dependent on the ecosystem at hand. We also discovered a range of correlations between the genetic capacity of a microbiome to produce PK and NRP secondary metabolites, and specific physicochemical (pH and salinity) and taxonomic diversity (Chao1 and Shannon) descriptors. Ultimately, we were able to accurately predict the PK biosynthetic potential of the microbial community at hand based on the system, the bacterial taxonomic richness, and the bacterial diversity.

Analysis of the microbial community composition revealed a high variability between the systems, and even between proximate sampling sites, highlighting the heterogeneity of microbiomes across small spatial scales. Whereas most of the measured environmental parameters seemed to have only a minor impact on the community, a notable exception being the strong correlation between pH and the bacterial taxonomic richness and community composition in soil. This corroborates previous findings of lower bacterial diversity in low pH soils (15, 16). By contrast, the fungal community, which is likely affected by pH to a lesser extend (24, 25) than the bacterial community due to a wider pH growth optimum (26), showed no significant correlation between richness and pH. A major driver of shifts in the sediment microbial communities is the availability of oxygen and, thus, the redox potential (27). Surprisingly, we observed that sediment microbiomes were largely similar across strong redox gradients and, hence, the distance between proximate sampling sites had a stronger clustering effect on the microbial community. This might be explained by the tide destabilizing microbial stratification in the coastal sediment, or the presence of stronger driving parameters that we failed to measure, e.g., difference in the macro fauna and flora. For the aquatic system, we observed a significant shift in bacterial and fungal community composition along a salinity gradient, corroborating the observation that salinity is a strong driver of community composition in estuarine waters (28, 29). In general, bacterial species richness scales with bacterial cell abundances across orders of magnitude (30), and while we did observe the highest species richness in the most densely populated system, i.e., soil, the observed abundances, which spanned 2 orders of magnitude, was not significantly correlated with changes in taxonomic richness.

Cross-system comparisons of biosynthetic diversity using publicly available amplicon sequencing data is hampered by the fact that most studies have focused on soil microbiomes, and, moreover, such comparisons are vulnerable to differences in experimental approaches across studies. Previous attempts at comparing marine and terrestrial systems have shown that the NRP and PK biosynthesis potential of these systems differ, and that the richness of KS and AD OBUs in marine microbiomes are likely comparable to, or higher than that of, more commonly sampled soil systems (7). Here, we applied the same PCR reagents and bioinformatic pipelines, regardless of the system at hand, and found pronounced system-specific differences in KS and AD composition as well. We found the NRP and PK biosynthesis potential, i.e., the AD and KS richness, to be in the range of hundreds of unique OBUs (531 AD OBUs ± 158 and 281 KS OBUs ± 116), with soil being the most rich in different PKs, and the marine sediment most rich in different NRPs. These richness estimates are, however, significantly lower than previously reported estimates (7, 12, 31), which is due to more stringent cutoffs in the denoising and clustering of AD and KS sequencing reads in the current pipeline. The combination of highly degenerated primers, multiple amplification cycles, and different stringencies in data processing, is likely responsible for the large variation in OBU richness often observed using identical primers and similar environmental samples (from 500 to over 7000 OBUs per sample; [10, 12, 21, 22]). Furthermore, this may cause an overestimation of diversity as well, which is why we chose to apply a more conservative approach. Despite this more conservative approach, we found that the majority of OBUs were absent from the MiBIG database, highlighting the potential for structural novelty across systems, and in marine sediments in particular.

The interconnection between the biosynthetic potential and the taxonomic composition of natural microbiomes has been the focus of several culture dependent and culture independent studies (11, 32, 33). In particular, the Actinobacteria have historically been recognized for their extensive biosynthetic capacity (34). To determine which taxa are descriptive of microbiomes with a high KS diversity, we fit a generalized linear model to the taxonomic data, and found that for KS diversity, relatively few zOTUs could explain the differences observed, and, hence, few bacterial taxa are potentially driving the collective KS biosynthesis potential of the microbiomes. Of the highly associated taxa, there was a notable underrepresentation of the Actinobacteria compared to acidobacterial taxa, represented with a positive association in the model (Table S2). Although such extrapolations should be interpreted with caution, our observations are substantiated by recent metagenomic analyses of soil microbiomes where the Acidobacteria, Verrucomicobia, Gammatimonadetes, and the candidate phylum Rokubacteria have been shown to carry a substantial fraction of the biosynthetic capacity (9, 32). Thus, increasing the focus on these taxa in bioprospecting efforts could increase chances of revealing novel bioactive PK compounds. The NRP biosynthesis potential is likely diluted across a wider collection of taxa, as the model included close to 50 bacterial taxa to cover 90% of the AD diversity. Again, most of the highly associated bacterial taxa were within the proteobacterial and acidobacterial phyla, followed by the Bacteroidetes and the Actinobacteria.

By investigating proximate microbiomes, we sought to eliminate major impacts due to dispersal limitation, yet distance remained an important factor for the diversity of biosynthetic genes in the microbiomes. Clearly, dispersal limitation and events of a stochastic nature are important factors involved in diversification of the secondary metabolome of microbial communities, but still we were able to identify environmental variables that had a significant influence on their biosynthetic capacity. Across the 3 systems, we found significant correlations between the capacity to produce PKs and pH, suggesting that within a pH range of 5–8, the richness of PKs increases with decreasing pH. In contrast, a positive effect of pH on the biosynthetic potential was observed for the soil system alone, and corroborates previous findings from similar terrestrial environments (10, 12, 21), though it has not been consistently observed (11). Similarly, low moisture content has been associated with increased biosynthetic diversity in different soil microbiomes (10). This association is, however, complex as expression of diverse BGCs increase in soil systems upon wetting, and consequently increasing available organic carbon content (32). Adding to this complexity, we found that moisture had significant, yet opposing, interdependencies with the capacity of the microbiomes to produce NRPs and PKs. Furthermore, the relative influence of moisture is skewed by the fact that we include moisture across terrestrial and aquatic systems, and the effect is likely attributed to the differences between these systems. Across systems, NRPS diversity was also negatively correlated with organic carbon content, and hence it is possible that heterotrophic bacteria and fungi compete for the available organic carbon, employing NRPs biochemistry in interference competition (35–37). While the freshwater biome has recently been deemed the most promising ecosystem in terms of gene cluster family richness (33), we found that salinity was not only a strong driver of overall community composition, as it was also significantly and positively correlated with the secondary metabolite biosynthesis potential in the aquatic system, suggesting that at least PKs may be more diverse in seawater. Previously suggested correlations between biosynthesis potential and environmental parameters, such as potassium, calcium, and selenium (10), could not be replicated in this study and are, hence, likely site-specific.

The variation in secondary metabolite biosynthetic diversity among bacteria is tied to taxonomy (33) and, hence, a higher taxonomic diversity is likely to result in a higher biosynthetic diversity. Additionally, a more diverse arsenal of secondary metabolites might be advantageous in a more diverse, and likely more competitive, community. To date, only a correlation between AD domain diversity and bacterial diversity in soil has been observed (21), yet we were able to substantiate this notion as we observed a significant correlation between bacterial diversity and PK biosynthetic richness across the three ecosystems.

Integrating a subset of the variables in a generalized linear model allowed us to predict the KS richness in different biomes with a high degree of confidence. The best model was obtained using ‘habitat’, bacterial taxonomic richness, and diversity as variables, whereas the fungal diversity added little to the predictive power of the model. The fact that both Chao1 index values and Shannon diversity are strong predictors underscores the tight coupling of the biosynthetic capacity of a microbiome to the total number of bacterial taxa, as well as the distribution, or evenness, of said taxa. This suggests that environmental microbiomes exhibiting a large number of zOTUs are more likely to have a high PKS biosynthesis potential, given that no single zOTU is dominating the microbiome. Taxonomic alpha diversity measures, such as Shannon diversity and Chao1 richness estimates are available for numerous public data sets, supporting their utility in predictive models, such as the current one. By contrast, few physicochemical metadata are currently accompanying publicly available data sets, which highlights the need for standardized operating procedures for metadata collection and reporting.

Applying the model to the Earth Microbiome data suggests that grasslands are the most promising biome in terms of PK biosynthesis potential. It is likely that the dense rhizosphere in such environments foster interactions and competition among microorganisms, and, consequently, hosts microbiomes with elaborate secondary metabolomes. Previous reports have suggested that rhizospheres harbor specifically talented secondary metabolite producers (38, 39), including members of the Actinobacteria, which have been shown to be preferentially enriched in grassland biomes compared to proximate tree-covered soils (9). Marine systems were classified as one biome in the Earth Microbiome data included here, but is clearly covering a multitude of diverse niches, which also comes across in the spread of the predicted KS biosynthesis potential, highlighting that divergent marine microbiomes hold a large hidden potential for PK production. Furthermore, the secondary metabolite biosynthesis potential was distinct between terrestrial and aquatic systems, which further supports the notion that these environments hold great promise for structural novelty. It should be noted that we used sequencing data from three biomes to make a predictive model apply to a large collection of biomes in these analyses, and the results are likely most reliable for biomes similar to the three biomes included here, i.e., temperate forest soils, temperate marine coastal sediments, and temperate surface waters. To broaden the utility of predictive models in bioprospecting, future models should extend to more diverse environments, in order to increase confidence in the output even further.

### Conclusion.

We determined that different ecosystems harbor microbiomes with distinct biosynthetic capabilities, and that relatively few taxa are highly associated with these differences, especially for PK biosynthesis genes. We identified taxonomic richness and diversity, as well as physicochemical variables, such as pH in soil and salinity in aquatic systems to be strong drivers of the biosynthetic diversity. Integrating these drivers in a predictive model allowed us to determine that grassland rhizospheres are likely promising sites in terms of finding novel PK compounds, and that marine microbiomes display a large variation in PK richness across the system, which should be explored in greater detail.

## MATERIALS AND METHODS

### Cross-system sampling.

Samples of topsoil (3 cm) were collected in sterile 50 mL Falcon tubes from 2 sites in, and around, the pristine deer park, Dyrehaven (Site 1 [S1]: 55°47'17.1′'N 12°32'51.1′'E and Site 2 [S2]: 55°48'46.3′'N 12°33'33.4′'E). S1 and S2 were situated in mixed coniferous and deciduous forests separated by 2.85 km (Fig. 1B). At each site, triplicates of 6 samples were collected for analysis. Proximate microbiomes were sampled 2 meters apart along a 10 m transect (S1), and 3 meters apart along a 15 m transect (S2), respectively. The collected soil samples were frozen and stored at −80° C within 1 h after sampling.

Marine coastal sediment cores were retrieved using a Hydro-Bios 60 cm sediment corer from two sites (S4 and S5), separated by 75 m in the shallow estuarine bay area of Nivå Bay Strandenge bird sanctuary, Nivå Bay (S4: 55°55'42.6"N 12°31'23.6"E, S5: 55°55'43.2"N 12°31'27.8"E) (Fig. 1C) At each site, 5 replicate sediment cores were sampled and sediment material from 3 different depths were collected: an upper layer (0 to 2 cm), a middle layer (3 to 5 cm), and a lower layer (5 to 7 cm). Additionally, samples of the overlaying water columns were taken in triplicate at each of the 2 sites as described for surface water sampling below, and processed the following day.

Triplicate water samples were collected at 9 different sampling stations within, and around, the temperate estuary of Ringkøbing Fjord (Fig. 1D). Water was collected and stored in 2 L polycarbonate carboys rinsed thrice with sample water. Smaller volumes were collected in 50 mL Falcon tubes for determining inorganic nutrients and metal ion concentrations, and in 40 mL sterile glass bottles containing 60 μL of a 17% H_3_PO_4_ solution for the determination of carbon content.

### Environmental metadata.

For the measurement of pH, 2 g of soil from each sample was mixed with 15 mL of water (pH 7.7). The pH was then measured under continuous stirring with an ATAGO DPH-2 digital pH-meter. The pH of the water samples was measured with the same approach on site. The redox potential was measured in the sediment samples using the GR 175-BNC-L01 Greisinger ORP/Redox electrode on site. The salinity, oxygen concentration, and temperature of the water were measured using a YSI Pro 2030 sensor.

Concentrations of phosphate and inorganic nitrogen compounds (nitrogen oxides, ammonium, and nitrite) were determined using segmented flow analysis (San++ System, Skalar Analytical BV). For the soil samples, 2 g of soil matrix was leached in 20 mL 2 M KCl solution overnight, shaking at 300 rpm. Subsequently, the leachate was filtered through 45 μm Minisart sterile filters (Sartorius), before the measurement, while the water samples were measured directly on the Skalar.

Ion chromatography (IC) was performed using an anion-exchange column equipped with a guard column (IonPac AS22, 4 × 250 mm, Ionpac AS22 Guard column, 4 × 50 mm, Thermo Scientific), and suppressed conductivity detection (suppressor Dionex AERS 500 for Carbonate, 4 mm, ThermoScientific, ICS-5000 Conductivity detector, ThermoScientific) was used to measure concentrations of fluoride, bromide, sulfate, and chloride. In order to measure concentrations of aluminum, calcium, iron, potassium, magnesium, sodium, phosphorus, sulfur, and selenium, the samples were analyzed by ICP-MS (7700x, Agilent Technologies) with Yttrium and Scandium as internal standards added directly in the samples. The instrument was equipped with Platinum tipped skimmer and sample cones, a double pass Scott spray chamber operated at 2° C, and a Micromist spray chamber. All elements were analyzed in He collision mode, with a Helium flow of 5 mL/min. Surface water and extracted sediment pore water were directly measured, while soil samples were pretreated by drying (60° C for 48 h) and leaching in 5 mL deionized water for 18 h.

The organic carbon content was estimated in both sediment and soil after initial drying, and subsequent measurement of loss on ignition (LOI) at 550° C. For the surface water samples, nonvolatile organic carbon (NVOC) content was determined by first bubbling nitrogen gas through the sample to eliminate the inorganic carbon. Then, sodium persulfate was added for oxidation, and the resulting carbon dioxide was measured by an infrared detector according with the Shimadzu Total Organic Carbon Analyzer TOC-Vwp with auto sampler ASI-V instructions.

Bacterial abundances were determined in all samples using SYBR Gold staining and fluorescence microscopy (Olympus BX51), as previously described (40). Surface water samples were fixed in 1% glutaraldehyde (final conc.), filtered, stained, and counted. For soil and sediment samples, 1 g of material was suspended in 9 mL of phosphate-buffered saline or 2% Sigma Sea Salt, respectively. Subsequently, bacterial cells were detached with sonication in a 2 × 50 W sonication bath at 28 kHz 3x30s. The suspensions were prefiltered with 5 μm polycarbonate membrane filters (GVS). Cells were captured on 0.2 μm Anodisc membrane filters (Whatman), stained, and counted.

The chlorophyll content was analyzed by filtering 250 to 500 mL of water through 5 μm SM membranes (Millipore). The filters were subsequently dissolved in 3 mL aqueous acetone solution, sonicated for 20 s, and incubated at 4° C in the dark overnight. The extract was then clarified by centrifuging for 20 min at 500 × *g*. Based on the absorbance at 630, 645, 663, and 720 nm, the chlorophyll a (Chl *a*) content was calculated according to established protocols (41). All collected metadata are listed in Table S1.

### DNA extractions.

Approximately 250 mg of thawed soil was transferred to a bead beating tube, and the DNA was extracted and purified using the PowerSoil kit from Qiagen according to the instructions provided by the manufacturer. The DNA was eluted twice in 50 μL elution buffer. Similarly, DNA was extracted from sediments using the PowerSoil kit, using 1000 mg of starting material, and eluting only once in 50 μL elution buffer. For surface water samples, biomass was collected by filtration of 50–250 mL of water onto 0.2 μm polycarbonate track etched filters (Whatman). The filters were subsequently submerged in 1.8 mL sucrose lysis buffer (400 mM NaCl, 750 mM sucrose, 20 mM EDTA, 50 mM Tirs-HCl, pH 8.5), and the DNA was extracted and purified using an established phenol-chloroform protocol (42).

### PCR amplification and sequencing.

To determine the bacterial and fungal taxonomic composition in the microbiomes, we amplified the V3V4 and the ITS region using established primer pairs (V3V4F_F 5′-CCTACGGGNGGCWGCAG, V3V4_R 5′-GACTACHVGGGTATCTAATCC, ITS_F 5′-GTGARTCATCRARTYTTTG, ITS_R 5′-TCCTSCGCTTATTGATATGC) (28, 43) tagged with an 8 bp sample specific barcode. The thermocycling conditions were 95° C for 15 min, followed by 30 cycles of 95° C for 30 s, 60° C for 30 s, 72° C for 30 s for V3V4, or 30 cycles of 95° C for 45 s, 50° C for 45 s, and 72° C for 90 s for ITS, and finally 72°C for 10 min for both V3V4 and ITS amplifications. The reaction mixtures contained TEMPase Hot Start Master Mix (Ampliqon), 0.32 μM each V3V4 primer, or 0.4 μM each ITS primer, and 0.3 μL or 2 μL template DNA per 25 μL reaction mix, respectively.

To assess the biosynthesis potential of the sampled microbiomes, we used an amplicon sequencing approach targeting conserved AD and KS regions, using the A3F-A7R and DegKS2F-R primer sets, as these have proven most broadly applicable (8). The AD regions in the samples were amplified using barcoded A3F – A7R primers (A3F 5′-GCSTACSYSATSTACACSTCSGG, A7R 5′-SASGTCVCCSGTSCGGTA) (44) under the following conditions: 15 min at 95° C, followed by 35 cycles of 94° C for 30 s, 56° C for 40 s, 72° C for 60 s, and finally 72° C for 10 min. For the surface water samples, 40 cycles instead of 35 cycles were performed in order to obtain sufficiently high DNA concentrations. The reaction mixtures contained HotStarTaq Master Mix (Qiagen), 0.32 μM each primer, 0.5 μL BSA, and 1 μL template per 25 μL reaction.

The KS region was amplified in a nested PCR. In the first round, amplification was done with the primers DegKS2F –DegKS2R (degKS2F 5′-GCNATGGAYCCNCARCARMGNVT, degKS2R 5′-GTNCCNGTNCCRTGNSCYTCNAC) (45), applying the same conditions as described for the AD PCR, with the exception of a slightly decreased annealing temperature of 55° C. The reaction mixtures contained HotStarTaq Master Mix (Qiagen), 1.92 μM each primer, 1 μL BSA, and 1 μL template per 25 μL reaction. The KS amplicons were subsequently purified with AMPure XP beads with a ratio of 1:1.2, and used as template for the PCR with barcoded primers: 95° C for 10 min, 15 cycles of 94° C for 30 s, 55° C for 30 s, 72° C for 45 s, and finally 10 min at 72° C. The reaction mixtures were the same as in the first PCR using 1 to 5 μL template.

All PCR products were cleaned up using AMPure XP beads with a ratio of 1:1.8 for the ITS amplicons, 1:1.4 for the V3V4 amplicons, and 1:1.2 for the KS and AD amplicons. The DNA concentrations were determined with the Invitrogen Quibit 2.0 fluorometer HS range kit, and the samples were pooled in equimolar ratios. Sequencing was performed by Novogene using PCR free library preparation and the NovaSeq PE250 system.

### Bioinformatic analysis.

The AD and KS reads were demultiplexed and brought into the same orientation using cutadapt v.3.4 (46). The primer sequence was trimmed off using seqtk v.1.3-r106 from the beginning of the reads (https://github.com/lh3/seqtk). For generating the OBUs, a combination of commands from USEARCH v.11.0.667 and Vsearch v.2.15.1 was used. First, the forward and the reverse reads were merged with an ‘N’. Subsequently, merged reads were error filtered (-fastq_maxee 5.0 accommodating the unmerged amplicons), dereplicated, and, finally, denoised using USEARCH unoise3 (47, 48). The resulting zOTUs were additionally clustered at 95% similarity to generate 4435 AD and 3381 KS OBUs, as has previously been performed (7) Lastly, the merged reads were mapped to the OBUs to generate the final abundance table. To determine and compare the degree of novelty of the biosynthesis potential in the 3 systems, the OBUs from each system was blasted against MiBiG v3.1, using a minimum alignment length of 100 nt, and an E-value cutoff < 10^−40^, as previously described (7).

Similarly, the processing of V3V4 and ITS reads consisted of a demultiplexing step using cutadapt, followed by merging of the forward and reverse reads. Subsequently, primers were removed, and the reads were brought into the same orientation with the option –revcomp in cutadapt. The reads were filtered for errors (- fastq_maxee 1.0), dereplicated, and denoised with unoise3, generating the zOTUs. The merged and clean reads were then mapped to the zOTUs, generating the abundance table. Taxonomy was assigned using usearch –sintax with rdp_16s_v16.fa and rdp_its_v2.fa as reference databases. Chloroplasts and zOTUs consistently present in negative control samples were removed from the V3V4 zOTU table. Average number of mapped reads per sample were 69402 ± 74736 and 90726 ± 100494 for V3V4 and ITS respectively, while they were 60253 ± 63865 and 32189 ± 41163 for AD and KS. Samples with less than 3000 mapped reads for AD, V3V4, and ITS or less than 1000 mapped reads for KS were excluded from the subsequent analysis, resulting in the exclusion of 8,9,3, and 7 samples, respectively. Rarefaction was performed to the same threshold, and the sample coverage estimated at the given threshold using the coverage function from the R package entropart (Fig. S5).

10.1128/msystems.00724-22.9 FIG S5 Rarefaction curves showing the coverage of AD, KS, V3V4, and ITS sequences and estimates of the average sample coverage (± SD) at the given cutoff (dotted line). Download FIG S5, EPS file, 2.3 MB. Copyright © 2023 Geers et al. 2023 Geers et al. https://creativecommons.org/licenses/by/4.0/ This content is distributed under the terms of the Creative Commons Attribution 4.0 International license.

### Statistical analysis.

For beta diversity analyses, the Bray-Curtis distance matrix was calculated from the relative abundances, excluding singletons. NMDS plots were made using the metaMDS function from the R package vegan. PERMANOVA tests on the distance matrix were used to identify significant grouping of samples using Adonis. The alpha diversity was assessed using the estimate_richness function in the R package phyloseq on the rarefied zOTU table, or OBU tables. To average out rarefying effects, the rarefication and calculation of richness/diversity was performed 500 times, and the final average taken. For all correlation analyses, the Pearson correlation coefficient was calculated, and the *P*-value computed using R and RStudio. Adjusted *P*-values were calculated using the p.adjust function in R with the Benjamini & Yekutieli correction for each dependent variable independently (Table S3).

10.1128/msystems.00724-22.3 TABLE S3 All calculated Pearson linear correlations of KS or AD richness, environmental variables, bacterial and fungal diversity, and their R and *P*-values. Download Table S3, XLSX file, 0.03 MB. Copyright © 2023 Geers et al. 2023 Geers et al. https://creativecommons.org/licenses/by/4.0/ This content is distributed under the terms of the Creative Commons Attribution 4.0 International license.

To predict the KS richness based on the bacterial community, a generalized linear model was used. The V3V4 zOTU table was pre-filtered removing zOTUs, retaining only those which had more than 0.5% relative abundance in at least 1 sample (550 V3V4 zOTUs). This meant that on average, 49.8% of the reads per sample were retained. Subsequently, the abundances were scaled using a centered log-ratio transformation, as described previously (20). The model was fitted using the glmnet R package with a lasso penalty term and Gaussian error distribution. The final lambda was selected, so that 90% of the deviation was explained. Generalized linear models were trained on 75% of the data using the glm function in R with a Gaussian error distribution and a log link function, followed by stepwise variable selection from both directions. The remaining 25% of the data was used to evaluate the performance of the model and compute the RMSE. The final model was applied to the Earth Microbiome data set, using the variables adiv_chao1, adiv_shannon, and empo_3 with the factors: Soil (non-saline), Water (non-saline), Water (saline), and Sediment (saline) as input. Additionally, the final model was applied to publicly available data, i.e., V3V4 reads from 3 seawater samples, for which KS reads were also obtained in a similar fashion as described in this paper (7), and the observed and predicted numbers of KS OBU were compared.

### Data availability.

Sequencing data are available in the Sequencing Read Archive (SRA) under bioproject PRJNA828401.
